# Extended genetic analysis and tumor characteristics in over 4600 women with suspected hereditary breast and ovarian cancer

**DOI:** 10.1186/s12885-023-11229-y

**Published:** 2023-08-10

**Authors:** Anna Öfverholm, Therese Törngren, Anna Rosén, Brita Arver, Zakaria Einbeigi, Karin Haraldsson, Anne Kinhult Ståhlbom, Ekaterina Kuchinskaya, Annika Lindblom, Beatrice Melin, Ylva Paulsson-Karlsson, Marie Stenmark-Askmalm, Emma Tham, Anna von Wachenfeldt, Anders Kvist, Åke Borg, Hans Ehrencrona

**Affiliations:** 1 Department of Oncology, Institute of Clinical Sciences, Sahlgrenska Academy, University of Gothenburg, Göteborg, Sweden; 2 Division of Oncology, Department of Clinical Sciences Lund, Lund University, Lund, Sweden; 3 Department of Radiation Sciences, Oncology, Umeå University, Umeå, Sweden; 4 Department of Oncology-Pathology, Karolinska Institutet, Stockholm, Sweden; 5 Department of Medicine and Oncology, Southern Älvsborg Hospital, Borås, Sweden; 6 Hereditary Cancer Unit, Karolinska University Hospital Solna, Stockholm, Sweden; 7 Department of Clinical Pathology and Clinical Genetics, Department of Clinical Experimental Medicine, Linköping University, Linköping, Sweden; 8 Department of Molecular Medicine and Surgery, Karolinska Institutet, Stockholm, Sweden; 9 Department of Clinical Genetics, Karolinska University Hospital, Stockholm, Sweden; 10 Department of Immunology, Genetics and Pathology, Uppsala University Hospital, Uppsala, Sweden; 11 Department of Genetics, Pathology and Molecular Diagnostics, Office for Medical Services, Region Skåne, Lund, Sweden; 12 Department of Clinical Science and Education at Södersjukhuset, Karolinska Institutet, Stockholm, Sweden; 13 Department of Oncology, Södersjukhuset, Stockholm, Sweden; 14 Division of Clinical Genetics, Department of Laboratory Medicine, Lund University, Lund, Sweden

**Keywords:** *BRCA1*, *BRCA2*, Genetic testing, Cancer, Breast cancer, Ovarian cancer, Hereditary cancer, Hereditary breast cancer, Hereditary ovarian cancer

## Abstract

**Background:**

Genetic screening for pathogenic variants (PVs) in cancer predisposition genes can affect treatment strategies, risk prediction and preventive measures for patients and families. For decades, hereditary breast and ovarian cancer (HBOC) has been attributed to PVs in the genes *BRCA1* and *BRCA2*, and more recently other rare alleles have been firmly established as associated with a high or moderate increased risk of developing breast and/or ovarian cancer. Here, we assess the genetic variation and tumor characteristics in a large cohort of women with suspected HBOC in a clinical oncogenetic setting.

**Methods:**

Women with suspected HBOC referred from all oncogenetic clinics in Sweden over a six-year inclusion period were screened for PVs in 13 clinically relevant genes. The genetic outcome was compared with tumor characteristics and other clinical data collected from national cancer registries and hospital records.

**Results:**

In 4622 women with breast and/or ovarian cancer the overall diagnostic yield (the proportion of women carrying at least one PV) was 16.6%. *BRCA1/2* PVs were found in 8.9% of women (*BRCA1* 5.95% and *BRCA2* 2.94%) and PVs in the other breast and ovarian cancer predisposition genes in 8.2%: *ATM* (1.58%), *BARD1* (0.45%), *BRIP1* (0.43%), *CDH1* (0.11%), *CHEK2* (3.46%), *PALB2* (0.84%), *PTEN* (0.02%), *RAD51C* (0.54%), *RAD51D* (0.15%), *STK11* (0) and *TP53* (0.56%). Thus, inclusion of the 11 genes in addition to BRCA1/2 increased diagnostic yield by 7.7%. The yield was, as expected, significantly higher in certain subgroups such as younger patients, medullary breast cancer, higher Nottingham Histologic Grade, ER-negative breast cancer, triple-negative breast cancer and high grade serous ovarian cancer. Age and tumor subtype distributions differed substantially depending on genetic finding.

**Conclusions:**

This study contributes to understanding the clinical and genetic landscape of breast and ovarian cancer susceptibility. Extending clinical genetic screening from *BRCA1* and *BRCA2* to 13 established cancer predisposition genes almost doubles the diagnostic yield, which has implications for genetic counseling and clinical guidelines. The very low yield in the syndrome genes *CDH1*, *PTEN* and *STK11* questions the usefulness of including these genes on routine gene panels.

**Supplementary Information:**

The online version contains supplementary material available at 10.1186/s12885-023-11229-y.

## Background

Over the past thirty years, genetic testing for inherited pathogenic variants in the *BRCA1* and *BRCA2* genes in patients with suspected hereditary breast and ovarian cancer (HBOC) has moved from research to being an established part of breast and ovarian cancer care. With studies demonstrating high cumulative cancer risks in affected women [1] and improved disease-specific and overall survival after risk-reducing surgery [2–4] the number of referrals for genetic analysis has steadily increased. Additionally, *BRCA1/2* status has clinical value as a treatment predictive marker, both for platinum-containing chemotherapy and for poly(ADP-ribose)polymerase inhibitors (PARPi) [5–8].

While mainstream testing of *BRCA1* and *BRCA2* has entered clinical care, it has become clear that the majority of women with presumed HBOC do not carry a pathogenic variant in either of these genes. The search for “*BRCA3*” has led to the conclusion that no gene with a similar population frequency of pathogenic variants and associated breast and ovarian cancer risk as *BRCA1* or *BRCA2* exists. Rather, the genetic landscape of predisposition to common cancers consists of very rare high-risk alleles in combination with common low-risk alleles (typically single nucleotide polymorphisms, SNPs) and an intermediate group of rare moderate-risk alleles [9]. Moderate-penetrance genes are defined as genes with risk alleles associated with odds ratio > 2 but ≤ 4 for risk of developing breast cancer, and high-penetrance as genes with risk alleles associated with odds ratio > 4 [10]. From an oncogenetic perspective, these groups all pose challenges for risk prediction and genetic counseling. Very rare syndromes such as Li-Fraumeni syndrome (*TP53*), Cowden/PTEN hamartoma syndrome (*PTEN*), Peutz-Jeghers syndrome (*STK11*) and hereditary diffuse gastric cancer and lobular breast cancer (*CDH1*) are all considered to be associated with high breast cancer risks [11], but due to the rarity of pathogenic variants and ascertainment bias in published studies, risk estimates are uncertain. The common SNPs are associated with too low cancer risks to be useful as individual markers in the clinical setting, but the combination of many such alleles into a polygenic risk score (PRS) has shown promise for cancer risk prediction both in the general population and as modifiers for carriers of high-risk variants [12–16].

Many candidate genes have been proposed to be associated with a moderate increased breast cancer risk, and with the introduction of the next generation sequencing (NGS) technology, several commercial laboratories began offering broad gene panels including genes with poorly defined risk. In 2012, the Swedish *BRCA1* and *BRCA2* study collaborators formed a national study named SWEA (The Swe-BRCA Extended Analysis) and designed a gene panel targeting 63 genes for use in the study. Established breast and/or ovarian cancer genes were included, at the time mainly *BRCA1*, *BRCA2* and a few syndrome genes, but also a long list of candidate genes for which rare alleles could potentially be associated with increased risk of developing breast cancer based on, for example, their functional role or published associations. Only pathogenic and likely pathogenic variants in established risk genes were reported back to the clinicians.

Only recently has the question of moderate-penetrance breast cancer risk genes largely been settled. In two large case–control studies published in 2021, a significant association with breast cancer was shown for protein truncating variants (PTVs) in *ATM, BARD1, BRCA1, BRCA2, CHEK2, PALB2, RAD51C* and *RAD51D*. Pathogenic variants in *TP53* only showed a significant association in the BRIDGES study [17], *CDH1* was associated only with estrogen receptor positive breast cancer in the CARRIERS study [18], *PTEN* failed to show significant association in either study and *STK11* was not associated with breast cancer in the BRIDGES study. These results illustrate the challenge to define risks for the very rare syndrome genes even in large case–control studies.

*BRIP1* is not established as a risk factor of clinical relevance for breast cancer but has previously been shown to be associated with a moderate increased risk for ovarian cancer [19–21].

In this article, we report our findings of pathogenic variants in the 13 established cancer predisposition genes *ATM, BARD1, BRCA1, BRCA2, BRIP1, CDH1, CHEK2, PALB2, PTEN, RAD51C*, *RAD51D, STK11* and *TP53* in a nation-wide cohort of over 4600 women with breast- and/or ovarian cancer. We also correlate the genetic findings with clinically relevant parameters such as age at diagnosis, tumor location, histopathological and molecular subtypes.

## Methods

### Healthcare setting

Assessment, counseling, and genetic testing for hereditary cancer is centralized to oncogenetic clinics at the six healthcare regions with university hospitals in Sweden. This service is reimbursed by the public health care system and accessible to all citizens on a national level. Patients with suspected hereditary breast and/or ovarian cancer fulfilling national criteria for genetic analysis of *BRCA1* and *BRCA2* (Additional file 1: HBOC genetic testing criteria in Sweden 2012–2018) were offered genetic testing. For the duration of this research study, clinical testing was centralized to one laboratory serving all healthcare regions (BRCAlab, Lund University, Lund, Sweden).

### Study cohort

The nation-wide SWEA study was open for inclusion between April 2012 and April 2018. Patients 18 years or older who were able to understand the written study information and where genetic screening of *BRCA1* and *BRCA2* was clinically indicated were offered inclusion in the study.

### Genetic analysis

All laboratory analyses were performed at the BRCAlab at the Department of Clinical Sciences at Lund University, Lund, Sweden. Targeted sequencing libraries were prepared from germline DNA extracted from blood using an Agilent SureSelectXT custom hybrid selection assay. The assay targeted 63 genes, including the 13 established cancer susceptibility genes that were the focus of this study (Additional file 1: Supplementary Methods). Apart from repetitive and low complexity genomic regions that are difficult to capture efficiently, the complete gene region of the 13 genes were captured by the assay, including introns and up- and downstream regions (Additional file 2: Table S9). Libraries were paired-end sequenced on an Illumina HiSeq 2000 or 2500 system to a target average sequence depth of 400–500 × over the targeted region. Sequences were aligned to the human reference genome (GRCh37) and genetic variants, including larger structural variants, were identified using a combination of variant calling tools. Variant calling was tuned for sensitivity and all variants classified as pathogenic or likely pathogenic in the 13 genes were confirmed using Sanger sequencing to identify potential false positives and safeguard accuracy. If any sample had below 30 × sequence coverage for any genomic position within coding exons and 20 base pairs of adjacent introns, that region was also Sanger sequenced to ensure complete coverage of all genes for all samples. The *BRCA1* and *BRCA2* genes were also analyzed in all samples using Multiplex Ligation-dependent Probe Amplification (MLPA), to confirm deletions and duplications of one or more exons. For details about library preparation, sequencing, alignment, variant calling, confirmatory Sanger sequencing, MLPA and variant classification, see Additional File 1: Supplementary Methods. For the entire study duration, regular national tumor board sessions were organized to discuss difficult cases and to reach agreements on variant classification and inclusion of additional clinical grade genes as evidence emerged.

If not specified, variants classified as pathogenic (class 5) or likely pathogenic (class 4) are collectively named pathogenic variants (PVs) in the Results and Discussion.

### Clinical parameters

For all patients where genetic analysis was performed, data on cancer diagnoses (e.g., age at diagnosis, histopathology, and localization) was collected from the referring oncogenetic clinics. This information was later confirmed using data from the cancer registry at the Swedish National Board of Health and Welfare, where all cancer diagnoses since 1958 are registered. We also included tumor-specific data from national quality registries for ovarian and breast cancer with coverage since 2008. Overall, there was a high level of consistency between the three data sources, and remaining discrepancies were manually assessed and curated by one of the authors (HE).

Morphology and biomarker data was derived from quality registers and pathology reports. For breast cancer, the definition of estrogen and progesterone receptor positivity (ER + , PR +) was ≥ 10% positive tumor cells detected by immunohistochemistry (IHC). HER2 status was determined based on IHC first, and in case of a 2 + IHC score followed by in-situ hybridization [22]. When defining breast cancer molecular subtypes, we assigned subtypes according to current Swedish national breast cancer guidelines, but the ER + , HER2-, Nottingham Histologic Grade 2 (NHG 2) subgroup was not further divided into Luminal A-like/B-like due to lack of consistent high-quality data for Ki67 [23].

### Statistical analysis

To analyze the association between categorical variables, the Pearson chi-square test (two-tailed) was used. All analyses were conducted using the IBM SPSS statistical computing package (IBM Corp. Released 2017. IBM SPSS Statistics for Windows, Version 25.0. Armonk, NY: IBM Corp). In the gene specific tables in Additional file 2, patients with one or two pathogenic variants in the same gene were not distinguished since it was not always possible to determine monoallelic or biallelic status. In case of bilateral breast cancer, each cancer was analyzed as an independent event.

## Results

### Study cohort

**Fig. 1:**
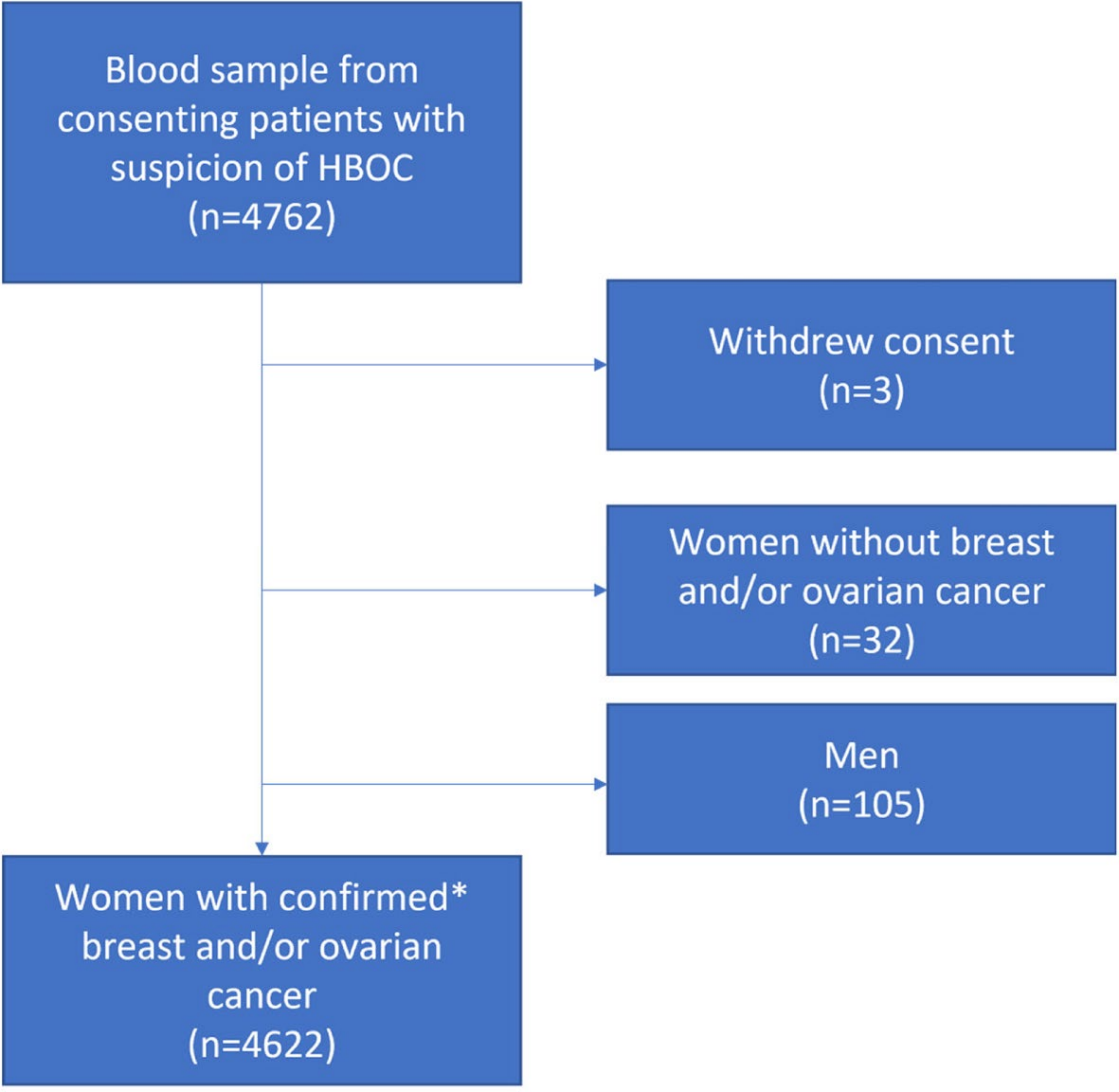
Flowchart summarizing the study cohort. *Cancer diagnoses confirmed against patient records including pathology reports, quality registries for breast and ovarian cancer, and/or the national cancer registry. HBOC = Hereditary breast and ovarian cancer

In total, blood samples from 4762 consenting individuals (97.8% women) with suspected HBOC were analyzed. Based on regional data at collaborating oncogenetic clinics and log files from BRCAlab, we estimate that these individuals represent about 85% of all patients screened for *BRCA1* and *BRCA2* in Sweden during the study period. Three women withdrew their consent after genetic analysis. Women without breast or ovarian cancer and men were not included in the main analysis, leaving a cohort of 4622 women with confirmed breast and/or ovarian cancer (Fig. 1). Of these, 4013 women had breast cancer only, 390 had ovarian cancer only, and 219 women had both breast and ovarian cancer. The median age for first breast cancer diagnosis was 45 years (range 21–87 years) and for ovarian cancer 57 years (range 20–90 years).

### Characteristics and classification of genetic variants

In this study we report 316 unique variants as pathogenic (*n* = 281) or likely pathogenic (*n* = 35) (Additional file 2: Table S1). Frameshift, nonsense, and canonical splice site variants predicted to cause loss-of-function, through nonsense mediated decay or disruption of critical protein functional regions, were classified as pathogenic or likely pathogenic. The pathogenicity of other variants was judged based on available evidence, such as amino acid conservation and biophysical properties, population allele frequencies and functional assays. Prior classifications and evidence of pathogenicity from the NIH-NCBI ClinVar archive (final accession 26 August 2022) and other databases and literature were considered. For *BRCA1* and *BRCA2,* we used the criteria defined by the Evidence-based Network for the Interpretation of Germline Mutant Alleles (ENIGMA; enigmaconsortium.org). The effect of variants predicted to alter splicing outside the canonical ± 1,2 positions was confirmed using cDNA sequencing and minigene assays (Additional file 2: Table S2). Due to uncertainty in the level of risk associated with missense variants in the moderate penetrance genes (*ATM, BARD1, BRIP1, CHEK2, RAD51C* and *RAD51D*), missense variants in these genes were not reported in this study.

No clinically relevant variants were detected in *STK11*. For the other genes, 32 large structural variants were detected (10.1% of all reported variants), and the other PTVs were classified as frameshift (142), stop gained (74) or as affecting splicing (46). Additionally, in *BRCA1*, *BRCA2* and *TP53*, 22 rare pathogenic and likely pathogenic missense variants were found. Out of the 316 variants, we classified 261 in concordance with previous ClinVar entries, and 52 variants were not found in ClinVar (Additional file 2: Table S1). The remaining 3 variants were missense variants in *TP53* with a status as variant of uncertain significance (VUS) in ClinVar. However, we have classified them as likely pathogenic (for further details, see Additional file 1: Discordant classification justification). The classification of all 316 variants has been submitted to ClinVar.

In total, 816 occurrences of the 316 unique PVs were reported in the entire study cohort (4759 individuals, Fig. 1). The frequency of PVs was skewed, where 215 unique variants (68.0%) were only identified once. On the other hand, some variants were commonly seen, with *CHEK2* c.1100del, p.(Thr367Metfs*15) being the single most frequent PV, reported in 142 individuals. 9 other variants were reported in 10 or more patients. Out of these, 7 were *BRCA1* variants known to be common in the Swedish population. The 2 remaining variants were *RAD51C* c.93del, p.(Phe32Serfs*8) and *BARD1* c.1690C > T, p.(Gln564*), seen in 13 and 11 individuals, respectively (Additional file 2: Table S1).

**Fig. 2:**
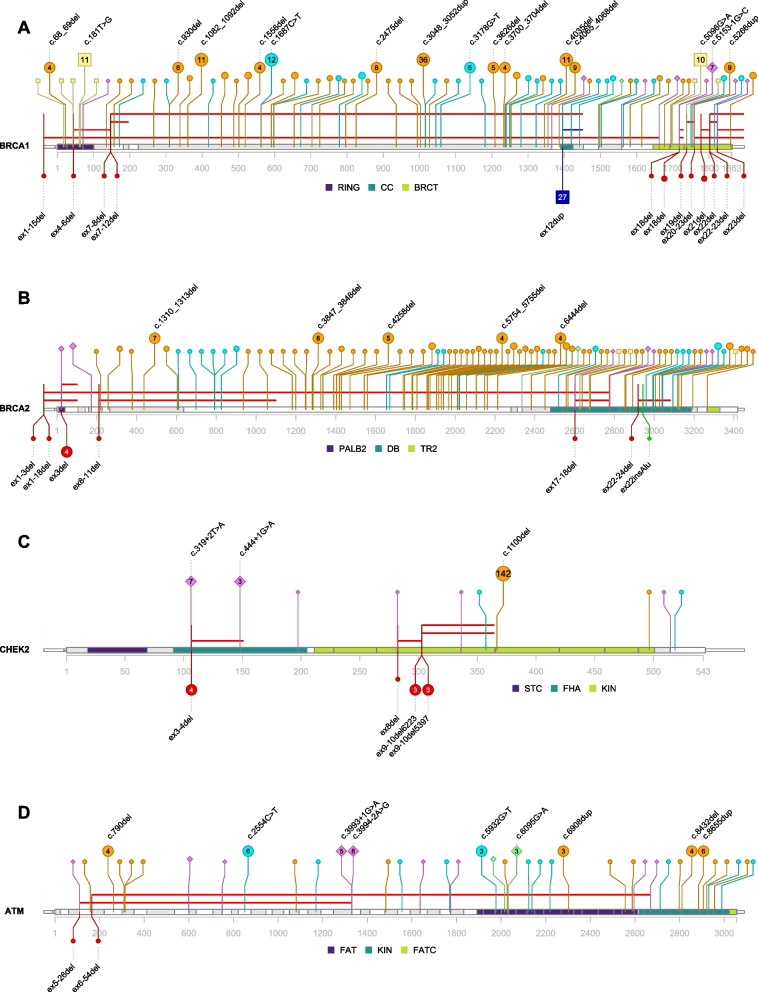
Lollipop plots showing the location and frequency of PVs in *BRCA1, BRCA2, CHEK2* and *ATM*. The gene model at the bottom of each plot shows exons in alternating shades of gray with untranslated regions thinner. Gene domains or other regions of interest are shown in colors defined by the legend below each gene model. The gene domain or region abbreviations are explained with references in Additional file 2: Table S6. The x-axis shows amino acid residue numbering according to the selected RefSeq protein for each gene. The number of carriers for each unique variant is indicated by the height and size of each lollipop and the inscribed number in the marker. The shape, color and location of the lollipops show the type of variant with structural variants below the gene model (red circle: large deletion, dark blue square: large duplication, green diamond: alu insertion) and other variants above (orange circle: frameshift, cyan circle: stop gain, pale yellow square: missense, purple diamond: intronic or synonymous splice variants, pale green diamond: exonic missense splice variant). Lollipops for variants in introns are placed at the border between adjacent exons. The horizontal bars above the gene model indicate the extent of large deletions (red) and large duplications (dark blue). All structural variants are labeled with a short form alias (the corresponding HGVS descriptions can be found in Additional file 2: Table S1) and the more common of the other variants are labeled with HGVS descriptions. Corresponding lollipop plots for *BARD1, BRIP1, CDH1, PALB2, PTEN, RAD51C, RAD51D* and *TP53* are shown in Additional file 3: Fig. S1

Six hundred sixty-nine (82.0%) of all detected PVs were found in the genes *BRCA1, BRCA2, CHEK2* and *ATM*, see Fig. 2 for visualization of these PVs in “lolliplot” graphs. Lolliplots for the other genes are depicted in Additional file 3: Fig. S1.

### Detailed assessment of variants in *TP53*

Evaluation of variants in *TP53* comes with some specific challenges (see Discussion). In this study, PVs in *TP53* were detected in 27 individuals, 1 man and 26 women. As detailed in Additional file 2: Table S3, segregation analysis in the families could confirm that the *TP53* variant was of germline origin in 14 individuals including the only man. In 4 women, the recurrent variant *TP53* c.542G > A, p.(Arg181His) was detected at near heterozygote variant allele frequency (VAF), consistent with probable germline origin. In our data, the 99% confidence interval for VAF for heterozygous carriers of variants of known germline origin was 40.1% to 59.9%. We therefore consider VAF within this range as consistent with a germline origin for heterozygous variants. Another group of 4 women had a personal and/or family history suggesting a probable germline variant, leaving 5 women with details either suggesting clonal hematopoiesis or uncertainty on the germline or somatic origin of the identified PV in *TP53*. One of these women had two concurrent PVs in *TP53* with a variant allele frequency at 29%, consistent with clonal hematopoiesis or somatic mosaicism.

### Individuals not included in the main analysis

Seventy-four participating men had breast cancer only, 18 had prostate cancer only, and 5 men had both breast and prostate cancer. PVs were detected in 17 of these men (17.5%), 2 in *BRCA1*, 8 in *BRCA2*, 3 in *ATM*, 3 in *CHEK2* and 1 in *TP53*. Thirty-two women and 8 men did not have a personal history of cancer motivating genetic testing, but typically were tested as presumed obligate carriers. Among these 40 individuals, three (7.5%) were heterozygous carriers of a PV in *BRCA1*.

### Diagnostic yield in women with breast and/or ovarian cancer

Seven hundred sixty-five of the 4622 women with breast and/or ovarian cancer carried at least one PV, for an overall diagnostic yield of 16.6%. As shown in Table 1, the group of women with both breast and ovarian cancer had the highest diagnostic yield (70/219, 32.0%), the ovarian cancer only group had a yield of 23.8% (93/390), and the lowest yield was seen in the breast cancer only group, 15.0% (602/4013).

**Table 1:** Diagnostic yield of pathogenic variants in subgroups of women with breast and/or ovarian cancer

	**Any variant class 4/5**^**a**^, n (%)	***P* Value**
***All women (n* = *4622)***	**765 (16.6)**	
***Diagnosis, breast and/or ovarian cancer (n* = *4622)***		*P* = 1.10 × 10^–13^
Breast cancer only (*n* = 4013)	602 (15.0)	
Ovarian cancer only (*n* = 390)	93 (23.8)	
Breast and ovarian cancer (*n* = 219)	70 (32.0)	
***Breast cancer, age at first diagnosis (n* = *4036)***		*P* = 0.0033
< 30 years (*n* = 186)	41 (22.0)	
30–39 years (*n* = 1055)	198 (18.8)	
40–49 years (*n* = 1360)	211 (15.5)	
50–59 years (*n* = 831)	124 (14.9)	
60–69 years (*n* = 463)	58 (12.5)	
≥ 70 years (*n* = 141)	17 (12.1)	
***Breast cancer, unilateral vs bilateral (n* = *4036)***		*P* = 2.01 × 10^–6^
Unilateral breast cancer (*n* = 3409)	508 (14.9)	
Bilateral breast cancer (*n* = 627)	141 (22.5)	
***First breast cancer, invasive vs *in situ* (n* = *3190)***		*P* = 0.28
Invasive breast cancer (*n* = 2932)	461 (15.7)	
In situ breast cancer only (*n* = 258)	34 (13.2)	
***Ovarian cancer, age at diagnosis (n* = *574)***		*P* = 0.00049
< 30 years (*n* = 21)	1 (4.8)	
30–39 years (*n* = 40)	7 (17.5)	
40–49 years (*n* = 104)	34 (32.7)	
50–59 years (*n* = 157)	55 (35.0)	
60–69 years (*n* = 169)	50 (29.6)	
≥ 70 years (*n* = 83)	11 (13.3)	
***Ovarian cancer, morphological subtype (n* = *485)***		*P* = 0.00030
Serous high grade (HGS) (*n* = 275)	99 (36.0)	
Serous low grade (LGS) (*n* = 48)	10 (20.8)	
Serous invasive not classified (*n* = 64)	14 (21.9)	
Clear cell (*n* = 25)	3 (12.0)	
Endometrioid (*n* = 24)	2 (8.3)	
Mucinous (*n* = 19)	3 (15.8)	
Borderline (*n* = 30)	3 (10.0)	
***Ovarian cancer, primary location (n* = *524)***		*P* = 0.49
Ovary (*n* = 387)	107 (27.6%)	
Fallopian tube (*n* = 72)	24 (33.3%)	
Peritoneal or abdomen NOS (*n* = 65)	16 (24.6%)	

In each subgroup, only cases with complete data for the respective variables were included. *P*-values calculated using the Pearson chi-square test (two-tailed)

^a^Sum of all detected variants in *PTEN, RAD51C, RAD51D* and *TP53*. For the corresponding tables subdivided by gene, see Additional file 2: Table S4a and S4b

*NOS* Not otherwise specified

The yield for *BRCA1* and *BRCA2* combined was 8.9%; 275 (5.95%) *BRCA1* and 136 (2.94) *BRCA2* carriers (Additional file 2: Table S4a). Very few variants were detected in the rare syndrome genes *CDH1* (0.11%, 5 carriers), *PTEN* (0.02%, 1 carrier) and *STK11* (no carriers detected in the entire cohort). PVs in *TP53* and *PALB2* were found in 26 (0.56%) and 39 (0.84%) women, respectively. For moderate penetrance genes, the highest yield was found in *CHEK2* with 160 (3.46%) carriers. Finally, 73 (1.58%) women had a PV in *ATM*, 21 (0.45%) in *BARD1*, 20 (0.43%) in *BRIP1*, 25 (0.54%) in *RAD51C* and 7 (0.15%) in *RAD51D*.

Most carriers had only one heterozygous PV, but 29 of the 765 women (3.8%) were identified as carriers of two different variants, and one woman carried three variants. Twenty-three of these women had variants in two different genes, whereas 7 women carried two variants in *CHEK2* (Additional file 2: Table S5). Interestingly, 6 of the 7 women with two *CHEK2* variants had bilateral breast cancer. Segregation analyses in the families could confirm that three of these women carried the *CHEK2* variants in *trans*, but for the remaining four women this information is lacking.

### Genetic findings in breast and ovarian cancer subgroups

The overall diagnostic yield of PVs in any investigated gene per subgroup of women is shown in Table 1 and per subgroup of breast cancer in Table 2. The corresponding yield per gene is displayed in Additional file 2: Table S4a and S4b.

**Table 2:** Pathogenic variants detected in breast cancer subgroups

	**Any variant class 4/5**^**a**^**, n (%)**	***P* Value**
***Breast cancer, NHG grade (n* = *2984)***		*P* = 2.00 × 10^–14^
NHG grade 1 (*n* = 401)	31 (7.7)	
NHG grade 2 (*n* = 1234)	154 (12.5)	
NHG grade 3 (*n* = 1349)	291 (21.6)	
***Breast cancer, morphological subtype (n* = *3376)***		*P* = 1.20 × 10^–9^
Ductal (*n* = 2735)	475 (17.4)	
Lobular (*n* = 301)	21 (7.0)	
Medullary (*n* = 61)	24 (39.3)	
Other invasive (*n* = 279)	42 (15.1)	
***Breast cancer, estrogen receptor status (n* = *3291)***		*P* = 2.38 × 10^–9^
ER positive (*n* = 2411)	345 (14.3)	
ER negative (*n* = 880)	203 (23.1)	
***Breast cancer, molecular subtype (n* = *2830)***		P = 6.54 × 10^–21^
ER + , HER2-, NHG 1 (Luminal A-like, *n* = 334)	25 (7.5)	
ER + , HER2-, NHG 2 (Luminal not classified, *n* = 859)	102 (11.9)	
ER + , HER2-, NHG 3 (Luminal B-like, *n* = 404)	97 (24.0)	
ER + , HER2 + (HER2-positive/Luminal, *n* = 430)	57 (13.3)	
ER-, HER2 + (HER2-positive/non-Luminal, *n* = 194)	24 (12.4)	
ER-, PR-, HER2- (Triple-negative, *n* = 609)	165 (27.1)	

In each subgroup, only cases with complete data for the respective variables were included. *P*-values were calculated using the Pearson chi-square test (two-tailed)

^a^Sum of all detected variants in *ATM, BARD1, BRCA1, BRCA2, BRIP1, CDH1, CHEK2, PALB2, PTEN, RAD51C, RAD51D* and *TP53*. For the corresponding tables subdivided by gene, see Additional file 2: Table S4a and S4b

*NGS* Nottingham Histologic Grade, *ER* Estrogen receptor, *PR* Progesterone receptor

The diagnostic yield was higher in women with younger age at breast cancer diagnosis and bilateral breast cancer (Table 1). A high yield was also seen in breast cancer cases with higher NHG grade and in the correlated subgroups medullary breast cancer, ER-negative breast cancer and triple-negative breast cancer (Table 2). As is evident from the gene-specific tables (Additional file 2: Table S4a and S4b) this result is largely driven by *BRCA1*.

In women with ovarian cancer, age at diagnosis and morphological subtype correlated with diagnostic yield (Table 1). As seen in the gene-specific table (Additional file 2: Table S4a), *BRCA1* PVs were most common in the age interval 50–59 years, whereas the highest frequency of *BRCA2* variants was seen at the age interval 60–69 years. The high diagnostic yield in women with high grade serous (HGS) morphological subtype was primarily due to PVs in *BRCA1*.

## Discussion

In this study, we report the results of genetic testing and clinical characterization of 4622 women with breast and/or ovarian cancer, referred for suspected HBOC. We found that 765 (16.6%) had at least one PV in any of the 13 genes that have solid evidence for a high or moderate increased risk of breast and ovarian cancer: *ATM, BARD1, BRCA1, BRCA2, BRIP1, CDH1, CHEK2, PALB2, PTEN, RAD51C, RAD51D, STK11* and *TP53* [17, 18, 24]. It should be noted that except for *TP53*, we detected very few PVs in the known rare syndrome genes *CDH1* (*n* = 5), *PTEN* (*n* = 1) and *STK11* (*n* = 0).

All oncogenetic clinics in Sweden participated in the study, and we estimate that this cohort represents about 85% of all women screened for PVs in HBOC-related genes in Sweden over the six-year inclusion period (2012–2018).

The clinical criteria for offering analysis of *BRCA1* and *BRCA2* during the study period were designed to roughly lead to a 10% diagnostic yield, and in accordance with that we found that 8.9% of the women had a PV in either of these two genes. In the other 11 genes, clinically relevant variants were detected in 8.2% of women, but since some carried more than one variant, the overall yield per woman was 16.6%. As shown in Tables 1 and 2, the overall yield was significantly higher in certain subgroups, e.g., women with both breast and ovarian cancer, women with high grade serous ovarian cancer, women with triple-negative breast cancer, or women with breast cancer at a younger age. At the same time, a reasonably high yield of 12.1% was seen even in the oldest subgroup of women with a breast cancer diagnosis at age 70 or older. Since most of the women are expected to have a family history for the disease (see “Genetic testing criteria” in Additional file 1), these figures are not generalizable to an unselected breast cancer population.

Recently, a follow-up of the BRIDGES breast cancer case–control study was published, where the molecular subtypes of tumors associated with 9 breast cancer predisposition genes (*ATM, BARD1, BRCA1, BRCA2, CHEK2, PALB2, RAD51C, RAD51D* and *TP53*) were reported [25]. While the numbers are relatively small in our study, it is interesting to note that the distribution of breast cancer molecular subtypes per gene (Additional file 2: Table S4b) shows a similar pattern as the association odds ratios presented in the BRIDGES follow-up study. In fact, for all 9 genes, the results between the two studies are completely consistent for the molecular subtypes with the highest mutation frequency per gene: PVs in *BARD1, BRCA1, RAD51C* and *RAD51D* were most common in triple-negative breast cancer, *ATM, BRCA2* and *PALB2* were enriched in the Luminal B-like subtype (ER + , HER2-, NHG 3), *CHEK2* was most frequently mutated in the ER + , HER2 + subtype, and *TP53* was enriched in HER2 + subtypes.

We identified 316 unique PVs. Most variant classifications were either concordant with existing ClinVar entries or PTVs that could easily be classified. For some variants we have performed additional analyses to clarify pathogenicity. Eighteen variants outside of canonical splice positions were shown to affect splicing (Additional file 2: Table S2). As an example, *PALB2* c.47A > C, p.(Lys16Thr) is a missense variant located in the last codon of exon 1 in *PALB2*, not previously reported to ClinVar. cDNA-analyses could confirm that the variant introduces a cryptic splice site, yielding a frameshift and a premature stop codon. We disagreed with ClinVar classifications on 3 missense variants in *TP53* (Additional file 1: Discordant classification justification), e.g., *TP53* c.328C > T, p.(Arg110Cys) has been reported on several occasions as a variant of uncertain significance (VUS) in ClinVar but was classified as likely pathogenic by us, partly based on functional characterization performed in another Swedish study [26].

Apart from missense variants that affect splicing, we only reported missense variants in the genes *BRCA1, BRCA2* and *TP53*. Especially for genes associated with a moderate increased risk for breast cancer, several reports show that missense variants as a group are associated with lower breast cancer risks than protein truncating variants in the same genes. For such missense variants, clinical utility is currently limited. One well-described example is *CHEK2* c.470 T > C, p.(Ile157Thr) that was identified multiple times in our study, but not reported due to its lower penetrance [17, 27]. It should be noted that there are exceptions to this rule, for instance the variant *ATM* c.7271 T > G, p.(Val2424Gly) acts in a dominant negative fashion and has been associated with higher risks than PTVs in the same gene [28–30]. *ATM* c.7271 T > G has a particularly high prevalence in Australia and was not identified in our study cohort.

In total, we detected 816 PVs in the entire cohort, and some variants were recurrent (Fig. 2, Additional file 3: Fig. S1, Additional file 2: Table S1). The most commonly detected PV was *CHEK2* c.1100del, p.(Thr367Metfs*15) that was seen 142 times, representing 83.5% of reported *CHEK2* variants or 17.4% of all variants. *CHEK2* was also the gene with the second highest frequency of PVs after *BRCA1* (Additional file 2: Table S4a). No individual had two PVs in *BRCA1*, consistent with the notion that except for cases with hypomorphic alleles, biallelic inherited loss of function of *BRCA1* is not compatible with survival [31–33]. On the other hand, *CHEK2* was the only gene in our study in which we did find double heterozygous variants (Additional file 2: Table S5). Biallelic loss of *CHEK2* has been associated with significantly higher breast cancer risks than heterozygote carriership in earlier studies [34, 35], and indeed, bilateral breast cancer was seen in six out of the 7 women with two PVs in *CHEK2*.

The assessment of variants in *TP53* is particularly challenging, since PVs in this gene have been shown to occur de novo including germline mosaicism [36] at a higher frequency than for other cancer predisposition genes, but it has also been demonstrated that such variants may have been acquired somatically in a process termed clonal hematopoiesis of indeterminate potential (CHIP) [37]. In Additional file 2: Table S3, we report our assessment of the 27 *TP53* carriers in this study, where we argue that it is likely that the majority carried a germline PV. A few cases were more uncertain, especially since recent findings suggest that even when the variant allele frequency is consistent with a heterozygous variant, a substantial proportion may be due to CHIP [38]. A special case was the only woman in this study who carried three variants, a heterozygous PV in *PALB2* and two *TP53*-variants at 29% VAF, strongly suggesting a somatic origin for both variants in *TP53* (Additional file 2: Table S3). Going forward, it may be advisable to analyze a follow-up skin biopsy for all patients with a presumed germline *TP53* PV detected in blood or saliva where segregation analysis in the family is uninformative.

Data sharing is important to improve genetic diagnostics, and we have submitted all classified pathogenic variants to ClinVar. Furthermore, we have shared data in international collaborations aiming to better define the risk associated with PVs in cancer predisposition genes. Through modified segregation analysis including families from this study, risk estimates for breast and ovarian cancer have been refined for PTVs in *RAD51C*, *RAD51D* [39], *PALB2* [40] and for the moderate penetrance missense variant *BRCA1* c. 5096G > A, p.(Arg1699Gln) (R1699Q) [41].

The strengths of this study include a large sample with nation-wide coverage, representative of clinical oncogenetic testing for suspected hereditary breast and ovarian cancer over several years. We achieved this by setting up a pragmatic study within the existing national oncogenetic network, in which all six participating clinics work in close collaboration. Hence, all patients fulfilling clinical criteria for routine genetic testing could be invited to the study. The centralized analyses at a national laboratory ensured quality and consistency in technical procedures and variant interpretation. Another strength is the detailed clinico-pathological annotation of cancer diagnoses from national registries, which have been shown to have a high degree of completeness [42–44]. With a study design where many candidate genes were sequenced but only validated clinically relevant variants were reported back (also retrospectively), we have avoided misinterpretation in the clinical setting of genetic variants that were considered interesting candidates a few years ago but now have been disputed, such as *RECQL* and *NBN* [17, 18]*.*

The study has some limitations. First, while the cohort is representative for oncogenetic clinics, the results cannot be extrapolated to unselected cancer cases or a healthy population. Second, even though there were national clinical criteria for when to offer analysis of *BRCA1* and *BRCA2*, we do not have detailed information on referral reasons for every single included individual. Third, we have not collected any data on ethnicity, preventing such subgroup analyses. Also, for the question of ovarian cancer predisposition it is a clear limitation that the genes for Lynch syndrome (*EPCAM, MLH1, MSH2, MSH6* and *PMS2*) were not included on the gene panel.

## Conclusions

The SWEA study contributes to an increased understanding of the genetic landscape of hereditary breast and ovarian cancer. Our results show that the addition of confirmed predisposition genes for breast and ovarian cancer almost doubles the diagnostic yield as compared with testing only for *BRCA1* and *BRCA2*. The preliminary results of this study have already informed national guidelines for cancer care. In the current Swedish national breast cancer guidelines [22] the syndrome genes with a very low frequency of findings (*CDH1, PTEN, STK11*) have been excluded from the routine clinical gene panel. All other genes reported in this article except *BRIP1* are now recommended for assessment of suspected hereditary breast cancer. *BRIP1* is instead included on the corresponding gene panel for ovarian cancer predisposition, together with *BRCA1, BRCA2, PALB2, RAD51C, RAD51D* and the Lynch syndrome genes [45].

### Supplementary Information

**Additional file 1.** HBOC genetic testing criteria in Sweden 2012-2018. Discordant classification justification. Supplementary methods.

**Additional file 2: Table S1.** Unique pathogenic/likely pathogenic variants detected in the Swedish cohort. **Table S2.** Splice site variants not affecting the canonical +- 1,2 basepairs. **Table S3.** Detailed assessment of *TP53* carriers in the Swedish cohort. **Table S4a.** Diagnostic yield of pathogenic variants per gene in subgroups of women with breast and/or ovarian cancer. **Table S4b.** Pathogenic variants per gene in breast cancer subgroups. **Table S5.** Women with two pathogenic/likely pathogenic variants. **Table S6.** Gene domains and regions depicted in lolliplots. **Table S7.** Sequences of adapters with 6 nucleotide long barcode sequences. **Table S8.** Sequences of adapters with 8 nucleotide long barcode sequences. **Table S9.** SureSelect custom hybrid selection assay design. **Table S10.** Primers for cDNA sequencing and minigene assays for analyses of splicing.

**Additional file 3: Figure S1.** Lollipop plots showing the location and frequency of PVs in *BARD1, BRIP1, CDH1, PALB2, PTEN, RAD51C, RAD51D* and *TP53*.

## Data Availability

All 316 pathogenic and likely pathogenic variants identified in this study, together with justification for classification in ambiguous cases, are available in the Additional files. The variant classifications have also been submitted to the database ClinVar. Datasets on clinical parameters and tumor characteristics are available from the corresponding author on reasonable request.

## Abbreviations

CHIP: Clonal hematopoiesis of indeterminate potential
ENIGMA: Evidence-based network for the interpretation of germline mutant alleles
ER: Estrogen receptor
HBOC: Hereditary breast and ovarian Cancer
IHC: Immunohistochemistry
MLPA: Multiplex ligation-dependent probe amplification
NGS: Next generation sequencing
NHG: Nottingham histologic grade
NOS: Not otherwise specified
PARPi: Poly(ADP-ribose)polymerase inhibitors
PR: Progesterone receptor
PRS: Polygenic risk score
PV: Pathogenic variant
PTV: Protein truncating variant
SNP: Single nucleotide polymorphism
Swe-BRCA: The Swedish *BRCA1* and *BRCA2* study collaborators
SWEA: The Swe-BRCA extended analysis study
VUS: Variant of uncertain significance
