# Supplementary material for: Extended genetic analysis and tumor characteristics in over 4600 women with suspected hereditary breast and ovarian cancer
Source: BMC Cancer. 2023 Aug 10;23:738. doi: 10.1186/s12885-023-11229-y (PMC10413543; doi:10.1186/s12885-023-11229-y)
Supplement: Supplementary file 1 — Additional file 1. HBOC genetic testing criteria in Sweden 2012-2018. Discordant classification justification. Supplementary methods. [file 12885_2023_11229_MOESM1_ESM.docx]

# Supplementary information

## HBOC genetic testing criteria in Sweden 2012-2018

## Discordant classification justification

## Supplementary methods

# A. HBOC genetic testing criteria in Sweden 2012–2018

- Breast cancer before age 35.
- Two cases of breast cancer among first-degree relatives*, one diagnosed before age 40. One of these cancers could also be ovarian cancer at any age.
- Three cases of breast cancer among first-degree relatives*, one diagnosed before age 50. One of these cancers could also be ovarian cancer at any age.
- Male breast cancer at any age.
- Two cases of ovarian cancer at any age.
- Breast and ovarian cancer in the same individual at any age.
- Minor criteria that strengthened the indication for testing were bilateral breast cancer and triple negative breast cancer, as well as prostate or pancreatic cancer.

** Female second-degree relatives included if on the paternal side.*

B. Discordant classification justification

***TP53* missense variants classified as likely pathogenic in this study but reported in ClinVar as variants of uncertain significance**

(ClinVar classifications retrieved 2022-08-26)

**NM_000546.5:c.328C>T, NP_000537.3:p.(Arg110Cys), p.(R110C)**

(ClinVar VariationID: 142206; Review status: criteria provided, multiple submitters, no conflicts).

This variant was found in one case in this study. It has also been observed in individuals with clinical features of Li-Fraumeni syndrome (ClinVar: Invitae), with *TP53* related tumors, but not classic Li-Fraumeni syndrome (ClinVar: Ambry) and with hereditary breast cancer (PMID: 31081129). The variant is rare in the gnomAD population database (v2.1.1: 5/251288 alleles; v3.1.2: 2/152172 alleles).

The variant affects a moderately conserved amino acid in the DNA-binding domain, beta sheet strand S1, replacing arginine with cysteine, a residue with substantially different physicochemical properties.

The residue is identified as a hotspot for recurrent mutations in human cancers with substitutions observed in 43 out of 24 592 tumor samples (cancerhotspots.org version 2, PMID: 29247016).

In-silico predictions do not provide evidence for or against pathogenicity for this variant (align-GVGD class C35, BayesDel score: 0.037; PMID: 29775997) and the variant is not predicted to have any impact on splicing (SpliceAI).

Yeast-based transactivation assays assessed on eight different promoters shows reduced transactivation activity (PMID: 12826609). Other studies using mammalian cells show no loss of growth suppression or dominant negative activity (PMID: 30224644, 29979965). A recent functional characterization of this variant show that it is associated with partial transactivation activity, decreased apoptotic activity, and cytoplasmic and perinuclear localization compared to wild type protein (PMID: 31081129), leading the authors to suggest a classification of likely pathogenic for this variant.

Five other amino acid substitutions at this codon are listed in ClinVar. Four show variant amino acids with moderately different properties compared to the wildtype arginine; two are classified as pathogenic or likely pathogenic (p.Arg110Pro, p.Arg110Leu) and two as uncertain significance (p.Arg110Gly, p.Arg110Ser). The fifth show a variant residue with similar properties to the wildtype and is classified as likely benign (p.Arg110His). The pathogenic classification of p.Arg110Pro indicates that this residue is important for *TP53* function. Based on the evidence shown above, we classify p.Arg110Cys as likely pathogenic.

**NM_000546.5:c.402T>G, NP_000537.3:p.(Phe134Leu), p.(F134L)**

(ClinVar VariationID: 1018290; Review status: criteria provided, single submitter).

This variant was found in a Li Fraumeni-like family in this study and segregates with early onset breast cancer (3 patients) and CNS tumor (1 patient). It has also been observed in a Li Fraumeni patient (PMID: 18685109) and patient(s) with breast and/or ovarian cancer (PMID: 29470806). The variant is absent in the gnomAD population database (v2.1.1; v3.1.2).

The variant affects a highly conserved amino acid in the DNA-binding domain, beta sheet strand S2 replacing phenylalanine with leucine, a residue with different physicochemical properties.

The residue is identified as a hotspot for recurrent mutations in human cancers with substitutions observed in 24 out of 24 592 tumor samples (cancerhotspots.org version 2, PMID: 29247016).

In-silico predictions do not provide evidence for or against pathogenicity for this variant (align-GVGD class C15, BayesDel score: 0.317; PMID: 29775997) and the variant is not predicted to have any impact on splicing (SpliceAI).

Yeast-based transactivation assays assessed on eight different promoters shows loss of transactivation activity (PMID: 12826609). Studies using mammalian cells show loss of growth suppression and dominant negative activity (PMID: 30224644, 29979965). Additional functional studies confirm that this variant is associated with loss of protein function (PMID: 31081129, 16322298, 16723121).

Two nucleotide variants causing the same amino acid substitution are reported in ClinVar. One (c.402T>A) is classified as pathogenic and uncertain significance, respectively, by two submitters providing classification criteria. The other (c.400T>C) is classified as uncertain significance.

Five other amino acid substitutions at this codon are listed in ClinVar. One shows a variant amino acid with large difference in properties to the wildtype phenylalanine and is classified as likely pathogenic (p.Phe134Cys), two show residues with moderately different properties to the wildtype and are classified as uncertain significance (p.Phe134Ser, p.Phe134Tyr). The final two show variant residues with smaller difference in properties to the wildtype and are classified as uncertain significance (p.Phe134Ile) or pathogenic and uncertain significance by two different submitters (p.Phe134Val). Based on the evidence shown above, we classify p.Phe134Leu as likely pathogenic.

**NM_000546.5: c.528C>G, NP_000537.3:p.(Cys176Trp), p.(C176W)**

(ClinVar VariationID: 376572; Review status: criteria provided, multiple submitters, no conflicts).

This variant was in a case with breast and ovarian cancer in this study. The variant is absent in the gnomAD population database (v2.1.1; v3.1.2).

The variant affects a highly conserved amino acid in the DNA-binding domain, loop L2A replacing cysteine with tryptophan, a residue with substantially different physicochemical properties. Together with three other residues, Cys-176 binds the structurally important zinc ion and is essential for stabilization of the protein core structure (PMID: 11793474).

The residue is identified as a hotspot for recurrent mutations in human cancers with substitutions observed in 115 out of 24 592 tumor samples (cancerhotspots.org version 2, PMID: 29247016).

In-silico predictions provide moderate evidence in support of pathogenicity for this variant (align-GVGD class C65, BayesDel score: 0.441; PMID: 29775997) and the variant is not predicted to have any impact on splicing (SpliceAI).

Yeast-based transactivation assays assessed on eight different promoters shows loss of transactivation activity (PMID: 12826609). Studies using mammalian cells show loss of growth suppression and dominant negative activity (PMID: 30224644, 29979965). Additional functional studies confirm that this variant is associated with loss of protein function (PMID: 19850740, 10753186).

Five other amino acid substitutions at this codon are listed in ClinVar. Four show variant amino acids with large differences in properties to the wildtype cysteine and are classified as pathogenic (p.Cys176Tyr), likely pathogenic (p.Cys176Phe), uncertain significance (p.Cys176Gly) or as pathogenic, likely pathogenic and uncertain significance by three different submitters (p.Cys176Arg). The fifth show a variant residue with moderate difference in properties to the wildtype and is classified as likely pathogenic and uncertain significance by different submitters (p.Cys176Ser). The pathogenic classification of p.Cys176Tyr indicates that this residue is important for *TP53* function. Based on the evidence shown above, we classify p.Cys176Trp as likely pathogenic.

# C. Supplementary methods

## DNA extraction

For the majority of the patients, DNA was extracted from peripheral blood lymphocytes using QuickGene-610, (Kurabo, Osaka, Japan). For other patients, extracted DNA was sent to the central laboratory from the oncogenetic clinics.

## Hybrid selection library preparation and sequencing

### DNA fragmentation

The concentration of extracted genomic DNA was measured by Qubit 1.0 (Invitrogen, CA, USA), using the Qubit dsDNA BR Assay Kit, and 1950 ng DNA was used as input to fragmentation. Fragmentation was performed on a Covaris S2 system (Covaris Inc, MA, USA), to a target average size of 300 bp (settings: time: 70 s, duty cycle: 7%, intensity: 4, cycles per burst: 200, set mode: frequency sweeping, temp: 4°C). The fragmented DNA was concentrated using 1.8x Agencourt AMPure XP beads (Beckman Coulter Genomics, Indianapolis, IN, USA) and eluted in 40 ul Elution Buffer, EB (Qiagen, Hilden, Germany). All AMPure XP bead clean-up steps were performed according to the manufacturer´s instructions and using appropriate bead input amount. Bead clean-up steps were run either manually or using KingFisherFlex (ThermoFisherScientific, Waltham, MA, USA).

### End repair and adaptor ligation

End-polishing, phosphorylation and adenylation were performed in one reaction as previously described (Neiman et al. 2012), with the following minor modifications: 34 ul fragmented DNA was mixed with 1x T4 DNA ligase buffer, 4 x 0.5 mM dNTP, 0.25 mM ATP, 0.12 U T4 DNA polymerase, 0.25 U T4 Polynucleotide kinase and 0.12 U Taq DNA polymerase (all enzymes and buffers from Fermentas Life Sciences, Burlington, Canada) and incubated for 20 min @ 25°C followed by 20 min @ 72°C. In-house designed adaptors, containing 6 or 8 nucleotide sample-specific barcodes (Additional file 2: Table S7 and S8), were used to make pre-capture pooling possible. 45 ul reaction mix from the enzymatic step was mixed with adaptors (final concentration of 2.2 uM), T4 DNA ligase (0.3 U) and T4 DNA ligase buffer (1x). Ligation reaction was carried out at 22°C for 30 min, then immediately put on ice.

### Size selection and enrichment of ligated fragments

One-sided size selection was performed with 0.7x AMPure XP beads to remove unligated adaptors and small fragments. PCR was run combining the eluted DNA, custom primers (forward 5’‑TGGAG TTCAG ACGTG TGCTC TTC‑3’, reverse: 5’‑CTTTC CCTAC ACGAC GCTCT TC‑3’) at a final concentration of 1 uM and 1x Phusion High-Fidelity PCR Master Mix (Finnzymes, Espoo, Finland). The volume was split in 4 x 50 ul reactions, and the PCR run at 98°C for 2 min, 6 cycles of 98°C for 20 s, 65°C for 30 s, 72°C for 30 s and a final elongation step at 72°C for 5 min. 1.1x AMPure XP beads were used to remove primers and nucleotides. DNA was eluted in 50 ul EB and concentration measured using Qubit (Qubit dsDNA HS Assay Kit).

### Hybrid selection target enrichment

The hybrid selection capture was performed using SureSelectXT Custom kit 3-5.9 Mb library (Agilent Technologies, Santa Clara, CA, USA). The SureSelect protocol Human All Exon Kit v1.0.1 was followed for the hybridisation setup and wash steps with the following modifications: eight samples were pooled before capture, 375 ng from each sample, and custom blockers (forward: 5’‑CTTTC CCTAC ACGAC GCTCT TCCGA TCT‑3’, reverse: 5’‑TGGAG TTCAG ACGTG TGCTC TTCCG ATCT‑3’) were added at a final concentration of 13.8 uM. PCR after capture was run using custom primers (PAGE purified, forward: 5‑AATGA TACGG CGACC ACCGA GATCT ACACT CTTTC CCTAC ACGAC GCTC‑3, reverse: 5‑CAAGC AGAAG ACGGC ATACG AGATG TGACT GGAGT TCAGA CGTGT GCTC‑3) at a final concentration 0.25 uM to add flow cell binding sequence to the library. The reaction was split into 4 x 50 ul and PCR was carried out at 98°C 30 s, 13 cycles of 98°C 10 s, 57°C 30 s, 72°C 30 s and a final elongation step at 72°C for 7 min. The PCR-product was purified using 1.0x AMPure XP beads. Agilent 2100 Bioanalyzer with the kit High Sensitivity DNA Kit (Agilent Technologies) was used to determine average size and the Qubit, Qubit dsDNA HS Assay Kit, to measure concentration.

### Hybrid selection custom assay design

The SureSelectXT Custom assay was designed to target the complete gene region for 17 genes, the coding exons and 20bp flanking introns for another 45 genes, and exon 2 of *CDK4* (Additional file 2: Table S9).

The tiling density of overlapping 120mer probes covering the genomic target regions was adjusted to compensate for GC/AT bias inherent in the library preparation. The effect of GC/AT bias was empirically estimated in two stages using two different development SureSelect custom assays. The first development assay was designed to target 16 complete genes (the same as the final design except *RAD51D*) using an even ~6x probe tiling (adjacent probes overlap by ~100bp). Based on eleven test libraries prepared from different samples using this assay, we calculated the mean normalized sequence coverage in 120mer sliding windows over the target genomic regions. Sliding windows were binned by genomic GC content and mean normalized coverage was calculated per GC bin as an estimate of the relative capture efficiency in relation to genomic GC content. The second development assay was designed against the same genomic target regions as the final design. Probe tiling density was adjusted based on the inverse of the capture efficiency for the GC-content bins estimated using the first development assay. The average probe tiling was ~3.5x, with 95% within a range from 1.5x to 12x tiling. Based on analysis of 173 test libraries prepared using this second design, 90 coding exons were identified that still had low coverage in some libraries and additional probes were added to boost coverage of these exons.

Target regions with high sequence similarity to other genomic regions can be hard to capture efficiently using hybrid selection assays as probes designed against these regions are likely to cause off-target capture (capture of non-target genomic regions). The extent of off-target capture will increase with increasing number of similar regions in the genome and their degree of similarity. Consequently, the efficiency with which the intended target is captured will also decrease. To identify probe sequences likely to yield high off-target capture, we extracted all possible 120-mer sequences from the target genomic regions and aligned them against the GRCh37 genome using cross_match version 1.090518 (http://www.phrap.org/phredphrapconsed.html) with default settings except -tags, -score_hist, -masklevel 0, -raw and -minscore 40. Potential probes with 30 or more matches in the genome with a cross_match score of 40 or higher or with 10 or more matches with a score of 60 or higher were excluded from use in the assay design. These cut-offs were based on analysis of the observed off-target capture using the first development assay and chosen to balance off-target capture, complete coverage of coding exons and broad coverage of the complete gene regions. The total length of intended design target genomic regions was 2 852 536 bp of which 78% was directly covered by the 56171 probes in the final assay design (for design metrics per gene, see Additional file 2: Table S9).

With a mean of 12.2 million read pairs sequenced per sample, the resulting mean coverage of the target region by high quality aligned reads was 385 and the mean coverage of the region covered by probes was 459 (discarding reads with mapping quality < 20 and bases with base quality < 20). On average, 92.7% of the complete target region and 99.86% of coding exons and 20 bp flanking introns of the 13 genes we focus on in this paper were covered by 30 reads or more. In 95% of all samples 89.8% of the target region and 99.42% of coding exons and flanking introns of the 13 genes was covered by 30 reads or more. For each sample, coding exon and flanking intron regions with coverage dipping below 30 reads in the 13 genes were Sanger sequenced to ensure complete coverage (see below). In 48.4% of samples there were no such regions of low coverage and 93.7% of samples had three or less regions with low coverage in the 13 genes (Additional file 2: Table S9). Recurrent regions of low coverage in *STK11* and *CDH1* accounted for 84% of samples with low coverage.

### Sequencing

Sequencing was carried out on an Illumina HiSeq 2000 or Illumina 2500 system in High Output or Rapid run mode (Illumina, PE-401-3001, FC-401-3001, PE-402-4001, FC-402-4001, PE-402-4002 and FC-402-4021). DNA fragments were paired-end sequenced with read lengths varying slightly over the course of the study between 93 bp + 94 bp up to 2 x 105 bp. The eight sample hybrid selection library pools were combined into a larger pool for sequencing of up to 48 barcoded sample libraries and spiked with 1% PhiX as quality control (Illumina, FC-110-3001). An aliquot was diluted to optimal concentration for loading on one or more lanes of one or two sequencing flowcells aiming for an average sequence coverage of the targeted genomic regions of about 400x.

## Sequence analysis

### Base calling and demultiplexing

Basecalls were generated on instrument by Illumina Real Time Analysis software (RTA) and converted to sequences in bam format using illumina2bam release 0.04 (github.com/wtsi-npg/illumina2bam). The custom adapters introduce sample-specific barcode sequences into both the first and the second read. Sequences were separated into per sample bam files (demultiplexed) using BamIndexDecoder (settings: MAX_MISMATCHES=3 MIN_MISMATCH_DELTA=2 MAX_NO_CALLS=1, github.com/wtsi-npg/illumina2bam).

### Alignment

Sequences were aligned to the human reference genome GRCh37 with decoy sequences from the 1000 genomes project (ftp://ftp.1000genomes.ebi.ac.uk/vol1/ftp/technical/reference/phase2_reference_assembly_sequence/) using novoalign version 3.02.00 (Novocraft Technologies Sdn Bhd, Petaling Jaya, Selangor, Malaysia, http://www.novocraft.com/products/novoalign/). Single nucleotide substitution variant alleles in dbSNP version 37 with an allele frequency of 1% or more in 1000 Genomes Project phase 1 genotype data were added to the reference genome as ambiguity codes to reduce allelic bias in the alignment. The Novoalign genome index was created with a k-mer length of 14 (-k 14) and step size of 2 (-s 2). Novoalign was first run on a subset of 2 million reads from each sample to get an initial estimate of fragment length mean and standard deviation and a base quality calibration table (settings: -r R -# 2M -t 160 --Q2Off -i PE 325,95). Thereafter, novoalign was rerun on the full sample sequence data using the fragment length mean and standard deviation and the quality calibration table from the first run as initial values. Default settings were used except --softclip 40 and --Q2Off. Adapters were trimmed from the reads by novoalign. Picard MarkDuplicates version 1.102 was used to identify and mark duplicate read-pairs likely to represent PCR or optical copies of the same template DNA (default settings, broadinstitute.github.io/picard/). Local realignment was performed using Genome Analysis Toolkit version 2.7-4-g6f46d11 (GATK, McKenna et al. 2010, DePristo et al., 2011) RealignerTargetCreator (default settings except: -mismatch 0.015) and IndelRealigner (default settings except: -model USE_SW -LOD 3 -entropy 0.015 -maxReads 70000). Base quality score recalibration on the aligned bams was performed using GATK BaseRecalibrator and PrintReads excluding known polymorphic sites in dbSNP 137 from the GATK resource bundle version 2.8. Basic sequence and alignment quality control metrics were calculated using Picard CalculateHsMetrics. Read coverage of targeted regions was calculated using GATK DepthOfCoverage and summarized using custom perl scripts.

### Detection of genetic variants

Genetic alterations, including single nucleotide substitution, small insertions and deletions and larger structural variants of various types were identified using GATK UnifiedGenotyper, Manta v1.2.1 (Chen et al. 2016), XHMM v1.0 (Fromer et al. 2012), Melt v2.1.3 (Gardner et al. 2017) and an in-house copy number visualization tool. All variants affecting coding exons and 20 bp flanking introns in the 13 genes we focus on in this paper were reviewed in Integrative Genomics Viewer (IGV, Robinson et al 2011) and subject to confirmatory Sanger sequencing and/or Multiplex Ligation-dependent Probe Amplification (MLPA), if classified as pathogenic or likely pathogenic (see below).

Substitutions and smaller insertions and deletions were called using GATK UnifiedGenotyper (default settings except -stand_call_conf 50 -stand_emit_conf 10). GATK VariantFiltration was used to mark potential variant call artefacts with filter annotations (for SNPs: QD<2.0, MQ<40.0, FS>60.0, MQRankSum<-12.5, ReadPosRankSum<-8.0; for indels: QD<2.0, FS>200.0, ReadPosRankSum<-20.0; for explanation of annotations used for filtering, see gatk.broadinstitute.org). However, all detected variants, including those marked with filters, were reviewed in IGV and confirmed with Sanger sequencing if classified as pathogenic or likely pathogenic, as described above.

Manta uses read pair and split read evidence to detect structural variants. Manta was run with default settings except for minCandidateVariantSize = 50, minScoredVariantSize = 50 and minDiploidVariantScore = 0. Detected variants with a sum of ≤ 30 confidently mapped pair reads supporting the variant at the upstream plus downstream breakend were discarded.

XHMM uses read depth evidence to detect copy number variants (CNVs) over specified genomic target intervals. We defined the target intervals for XHMM as the target intervals for the hybrid selection assay split at regions with low coverage based on a set of 100 samples (>5% of samples with coverage <30x) or with low mappability (50-mer mappability of <1 where mappability is defined as 1/[number of matches found in the genome] allowing for up to 2 mismatches, Derrien et al 2012). The mappability track created by Thomas Derrien and Paolo Ribeca in Roderic Guigo's lab at the Centre for Genomic Regulation (CRG), Barcelona, Spain was downloaded from UCSC genome browser. Our rationale for defining XHMM target regions in this manner is that Manta will discover structural variants with high sensitivity in regions well covered by high quality aligned reads and the copy-number based detection by XHMM is only needed for variants with break-ends in regions with low coverage or low-quality alignments. XHMM was run with default settings except --minTargetSize 50 --maxTargetSize 50000 --minMeanTargetRD 20 --maxMeanTargetRD 2000 --minMeanSampleRD 10 --maxMeanSampleRD 1000 --maxSdSampleRD 400 and --maxSdTargetRD 60. Detected variants < 51 nt and < 999 999 nt long or with non-diploid Q score of 50 or less were discarded.

Melt detects transposable element insertions based on discordant read pairs where one read maps to the reference genome and one read maps to a reference mobile element sequence. Melt was run with included priors from the 1000 genomes project (Altshuler et al. 2015, Sudmant et al. 2015). Average coverage, read length and insert size was calculated and set as parameters for each sample individually. Minimum contig size for calling elements was set to 50 bp, -exome flag was set, and we increased maximum reads in memory to 50000.

Large deletions and duplications were also detected from sequence read depth using in-house developed copy number visualization tool that computes a normalized read depth ratio between a sample and an average of multiple normal samples (baseline) in sliding windows over the target region. Read depth is GC-normalized before computing the ratio. Regions with normalized read depth ratio below 0.75 or above 1.25 are flagged as potential deletions or duplications, respectively.

### Variant effect annotations

The Ensembl Variant Effect Predictor (VEP, McLaren et al 2016) version 101 was used to annotate the predicted functional impact of a variant on a transcript and protein. Splice site consequences were predicted using the VEP plugins SpliceRegion and MaxEntScan (Shamsani et al. 2018). HGVS variant nomenclature was validated using VariantValidator (Freeman et al 2018).

## Variant pathogenicity classification

Frameshift, nonsense, and canonical splice site variants predicted to cause loss-of-function for disease-relevant transcripts, through nonsense mediated decay or disruption of critical protein functional regions, were classified as pathogenic or likely pathogenic (Abou Tayoun et al. 2018). Accordingly, variants conferring stop codons C-terminal of 55 nucleotides from the end of the next-to-last exon, although not predicted to cause NMD, can be classified as pathogenic or likely pathogenic if a critical protein function is disrupted. For example, nonsense variant c.9924C>G (p.Tyr3308Ter) in the last exon of *BRCA2* is pathogenic while c.9976A>T (p.Lys3326Ter) and nonsense variants C-terminal thereof are not considered pathogenic. Pathogenicity of other variants, including missense variants, short in-frame indels and variants predicted to alter splicing, were judged by evaluating available evidence similar to the American College of Medical Genetics guidelines (Richards et al. 2015) and, for *BRCA1* and *BRCA2*, according to criteria defined by the Evidence-based Network for the Interpretation of Germline Mutation Alleles (ENIGMA; enigmaconsortium.org; submit.ncbi.nlm.nih.gov/ft/byid/vuhooppz/enigma_rules_2017-06-29-v2.5.1.pdf). Evidence considered include amino acid conservation and biophysical properties, predicted effect on splicing, segregation, population and case-control allele frequencies, family history, pathology, and functional assays. Prior classifications and associated evidence in ClinVar were also considered. For the moderate penetrance genes (*ATM, BARD1, BRIP1, CHEK2, RAD51C* and *RAD51D*), missense variants were not reported in this study. For variants near splice sites (3 nt of the exon and 20 nt of the intron adjacent to each intron/exon border) but outside the canonical +/- 1,2 positions, predicted by MaxEntScan (Yeo and Burge, 2004, Vallée et al. 2016) to alter splicing, and for which convincing published experimental evidence was not available, we performed complementary DNA (cDNA) sequencing and/or reporter minigene assays to confirm variant effect on splicing (see below). Intronic variants more than 20 nt from the nearest donor or acceptor exon/intron border were not considered for variant pathogenicity classification, implicitly classifying them as variants of uncertain significance, likely benign or benign for the purposes of this paper.

## Multiplex Ligation-dependent Probe Amplification (MLPA)

Multiplex Ligation-dependent Probe Amplification (MLPA) was run according to the manufacturer´s instructions (MRC-Holland, Amsterdam, The Netherlands) and analysed using GeneMarker v1.6 (Softgenetics, PA, USA). Clinical samples were analysed for *BRCA1* (SALSA MLPA Kit P002) and *BRCA2* (P045). P087 (*BRCA1*) and P090 (*BRCA2*) were used to confirm variants detected using MLPA.

## Variant confirmation using Sanger sequencing

Variants, including larger structural variants, classified as pathogenic or likely pathogenic in the 13 genes were all confirmed with Sanger sequencing to identify potential false positives and ensure accuracy. Sanger sequencing was performed with Big Dye Terminator v3.1 Cycle Sequencing Kit (Applied Biosystems, Foster City, CA, USA) and ran on a 3130XL Genomic Analyzer, Applied Biosystem. Sequencher® version 5.0 DNA sequence analysis software (Gene Codes Corporation, Ann Arbor, MI USA) was used to evaluate the chromatograms.

## Sanger sequencing of low coverage regions

If any sample had below 30x sequence coverage for any genomic position within coding exons and 20 base pairs of flanking introns in any of the 13 genes we focus on in this paper, a new SureSelect library was prepared and sequenced or the regions with low coverage were Sanger sequenced as described above to ensure complete coverage of all genes for all samples.

## Splice variant analysis using cDNA sequencing and minigene-based assays

The effect of variants predicted to alter splicing outside the canonical +/- 1,2 positions, was investigated using cDNA sequencing and/or minigene assays.

For cDNA sequencing, whole blood from patients carrying the splice variant was collected in Tempus Blood RNA Tubes (Applied Biosystems, CA, USA) and RNA was extracted with Tempus Spin RNA Isolation Reagent Kit. Total RNA was reverse-transcribed into cDNA using SuperScript IV according to manufacturer´s instructions (Invitrogen, Waltham, MA, USA). Regions of the transcripts likely to be affected by the variant under investigation were amplified from cDNA (primers in Additional file 2: Table S10) and Sanger sequenced as described above.

Reporter pCAS minigene assays were performed as described in Bonnet et al 2008 and Tournier et al 2008. Briefly, relevant exons and about 150 nucleotides of adjacent introns were amplified from genomic DNA of patients carrying the splice variant using primers containing 5’ tails with BamHI and MluI restriction sites (Additional file 2: Table S10). The amplified products were inserted into the pCAS vector utilizing the corresponding restriction sites of the vector. By sequencing inserts, wild-type and variant allele minigene constructs were selected for transient transfection into HeLa cells. In cases where we had previously performed minigene assays for the same relevant exon, we re-used the pCAS wildtype construct and created the variant allele by site-directed mutagenesis using QuikChange II Site-Directed Mutagenesis Kit according to the manufacturer´s instructions (Agilent). The insert was sequenced to verify the variant allele. RNA extracted from transfected cells was reverse-transcribed and amplified using primers complementary to the pCAS minigene exons. The cDNA products of minigenes carrying the variant and the wild-type alleles were then Sanger sequenced as described above to identify splicing patterns.

## Supplementary Methods References

Neiman M, Sundling S, Grönberg H, Hall P, Czene K, Lindberg J, et al. Library preparation and multiplex capture for massive parallel sequencing applications made efficient and easy. PLoS One. 2012;7(11):e48616.

McKenna A, Hanna M, Banks E, Sivachenko A, Cibulskis K, Kernytsky A, et al. The Genome Analysis Toolkit: a MapReduce framework for analyzing next-generation DNA sequencing data. Genome Res. 2010 Sep;20(9):1297-303.

DePristo MA, Banks E, Poplin R, Garimella KV, Maguire JR, Hartl C, et al. A framework for variation discovery and genotyping using next-generation DNA sequencing data. Nat Genet. 2011 May;43(5):491-8.

Chen X, Schulz-Trieglaff O, Shaw R, Barnes B, Schlesinger F, Källberg M, et al. Manta: rapid detection of structural variants and indels for germline and cancer sequencing applications. Bioinformatics. 2016 04 15;32(8):1220-2.

Fromer M, Moran JL, Chambert K, Banks E, Bergen SE, Ruderfer DM, et al. Discovery and statistical genotyping of copy-number variation from whole-exome sequencing depth. Am J Hum Genet. 2012 Oct 5;91(4):597-607.

Gardner EJ, Lam VK, Harris DN, Chuang NT, Scott EC, Pittard WS, et al. The Mobile Element Locator Tool (MELT): population-scale mobile element discovery and biology. Genome Res. 2017 11;27(11):1916-29.

Robinson JT, Thorvaldsdóttir H, Winckler W, Guttman M, Lander ES, Getz G, et al. Integrative genomics viewer. Nat Biotechnol. 2011 Jan;29(1):24-6.

Derrien T, Estellé J, Marco Sola S, Knowles DG, Raineri E, Guigó R, et al. Fast computation and applications of genome mappability. PLoS One. 2012;7(1):e30377.

Auton A, Brooks LD, Durbin RM, Garrison EP, Kang HM, Korbel JO, et al. A global reference for human genetic variation. Nature. 2015 Oct 1;526(7571):68-74.

Sudmant PH, Rausch T, Gardner EJ, Handsaker RE, Abyzov A, Huddleston J, et al. An integrated map of structural variation in 2,504 human genomes. Nature. 2015 Oct 1;526(7571):75-81.

McLaren W, Gil L, Hunt SE, Riat HS, Ritchie GR, Thormann A, et al. The Ensembl Variant Effect Predictor. Genome Biol. 2016 06 6;17(1):122.

Shamsani J, Kazakoff SH, Armean IM, McLaren W, Parsons MT, Thompson BA, et al. A plugin for the Ensembl Variant Effect Predictor that uses MaxEntScan to predict variant spliceogenicity. Bioinformatics. 2019 07 1;35(13):2315-7.

Freeman PJ, Hart RK, Gretton LJ, Brookes AJ, Dalgleish R. VariantValidator: Accurate validation, mapping, and formatting of sequence variation descriptions. Hum Mutat. 2018 01;39(1):61-8.

Abou Tayoun AN, Pesaran T, DiStefano MT, Oza A, Rehm HL, Biesecker LG, et al. Recommendations for interpreting the loss of function PVS1 ACMG/AMP variant criterion. Hum Mutat. 2018 11;39(11):1517-24.

Richards S, Aziz N, Bale S, Bick D, Das S, Gastier-Foster J, et al. Standards and guidelines for the interpretation of sequence variants: a joint consensus recommendation of the American College of Medical Genetics and Genomics and the Association for Molecular Pathology. Genet Med. 2015 May;17(5):405-24.

Yeo G, Burge CB. Maximum entropy modeling of short sequence motifs with applications to RNA splicing signals. J Comput Biol. 2004;11(2-3):377-94.

Vallée MP, Di Sera TL, Nix DA, Paquette AM, Parsons MT, Bell R, et al. Adding In Silico Assessment of Potential Splice Aberration to the Integrated Evaluation of BRCA Gene Unclassified Variants. Hum Mutat. 2016 07;37(7):627-39.

Bonnet C, Krieger S, Vezain M, Rousselin A, Tournier I, Martins A, et al. Screening BRCA1 and BRCA2 unclassified variants for splicing mutations using reverse transcription PCR on patient RNA and an ex vivo assay based on a splicing reporter minigene. J Med Genet. 2008 Jul;45(7):438-46.

Tournier I, Vezain M, Martins A, Charbonnier F, Baert-Desurmont S, Olschwang S, et al. A large fraction of unclassified variants of the mismatch repair genes MLH1 and MSH2 is associated with splicing defects. Hum Mutat. 2008 Dec;29(12):1412-24.
