# Supplementary figures and images for: Extended genetic analysis and tumor characteristics in over 4600 women with suspected hereditary breast and ovarian cancer
Source: BMC Cancer. 2023 Aug 10;23:738. doi: 10.1186/s12885-023-11229-y (PMC10413543; doi:10.1186/s12885-023-11229-y)

A

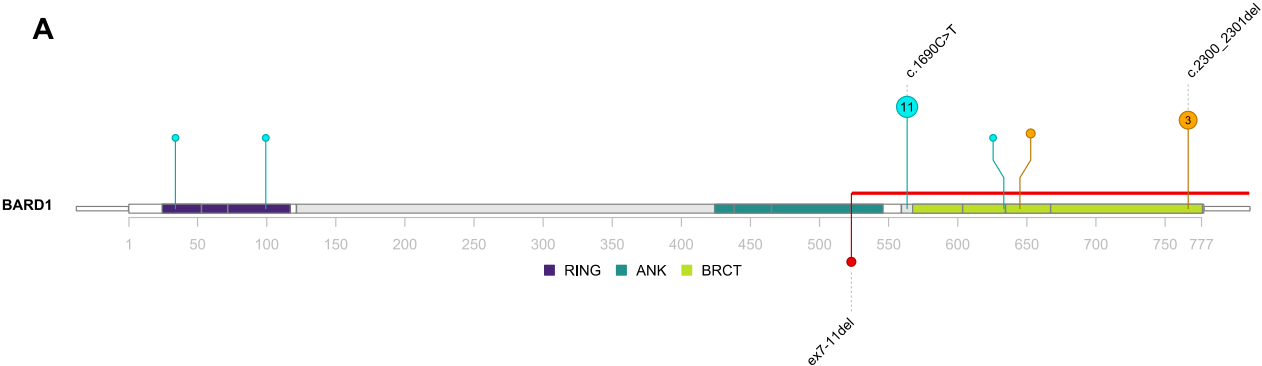

B

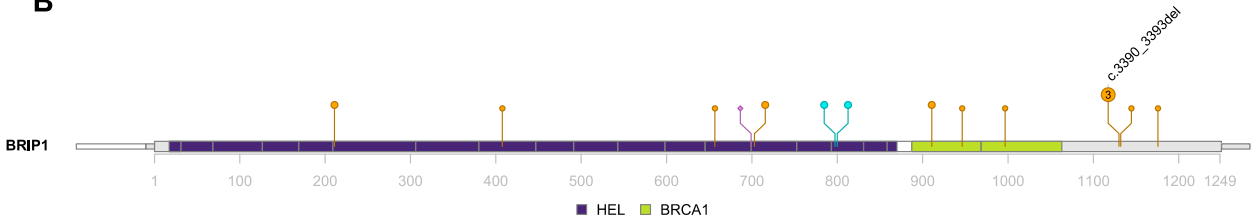

C

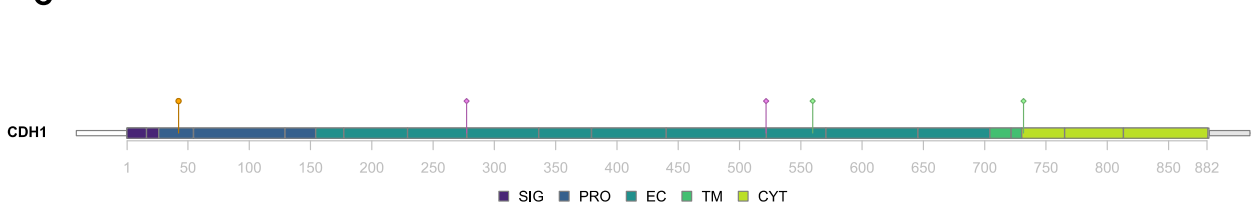

D

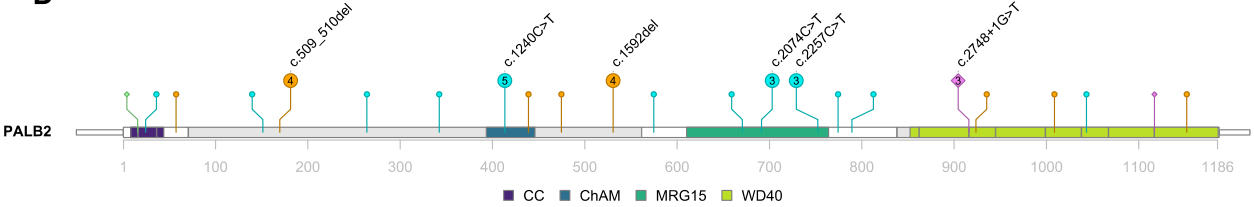

E

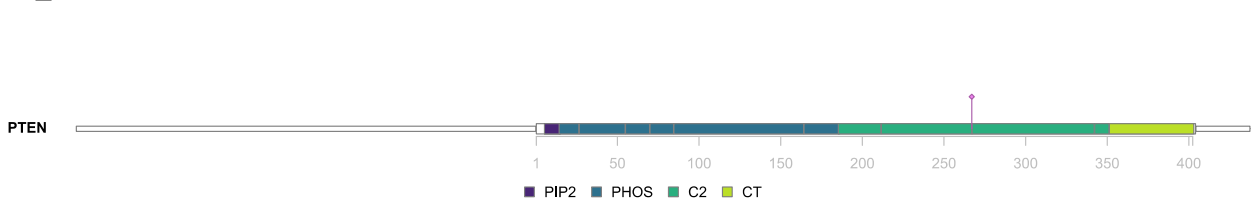

Supplement: Supplementary file 3 — Additional file 3: Figure S1. Lollipop plots showing the location and frequency of PVs in BARD1, BRIP1, CDH1, PALB2, PTEN, RAD51C, RAD51D and TP53. [file 12885_2023_11229_MOESM3_ESM.pdf]
